# PAGED: a pathway and gene-set enrichment database to enable molecular phenotype discoveries

**DOI:** 10.1186/1471-2105-13-S15-S2

**Published:** 2012-09-11

**Authors:** Hui Huang, Xiaogang Wu, Madhankumar Sonachalam, Sammed N Mandape, Ragini Pandey, Karl F MacDorman, Ping Wan, Jake Y Chen

**Affiliations:** 1 School of Informatics, Indiana University, Indianapolis, IN 46202, USA; 2 Indiana Center for Systems Biology and Personalized Medicine, Indiana University, Indianapolis, IN 46202, USA; 3 MedeoLinx, LLC, Indianapolis, IN 46280, USA; 4 Capital Normal University, Beijing, 100048, China

## Abstract

**Background:**

Over the past decade, pathway and gene-set enrichment analysis has evolved into the study of high-throughput functional genomics. Owing to poorly annotated and incomplete pathway data, researchers have begun to combine pathway and gene-set enrichment analysis as well as network module-based approaches to identify crucial relationships between different molecular mechanisms.

**Methods:**

To meet the new challenge of molecular phenotype discovery, in this work, we have developed an integrated online database, the Pathway And Gene Enrichment Database (PAGED), to enable comprehensive searches for disease-specific pathways, gene signatures, microRNA targets, and network modules by integrating gene-set-based prior knowledge as molecular patterns from multiple levels: the genome, transcriptome, post-transcriptome, and proteome.

**Results:**

The online database we developed, PAGED http://bio.informatics.iupui.edu/PAGED is by far the most comprehensive public compilation of gene sets. In its current release, PAGED contains a total of 25,242 gene sets, 61,413 genes, 20 organisms, and 1,275,560 records from five major categories. Beyond its size, the advantage of PAGED lies in the explorations of relationships between gene sets as gene-set association networks (GSANs). Using colorectal cancer expression data analysis as a case study, we demonstrate how to query this database resource to discover crucial pathways, gene signatures, and gene network modules specific to colorectal cancer functional genomics.

**Conclusions:**

This integrated online database lays a foundation for developing tools beyond third-generation pathway analysis approaches on for discovering molecular phenotypes, especially for disease-associated pathway/gene-set enrichment analysis.

## Background

Pathway analysis and gene-set enrichment analysis are both widely-used methods to identify significant molecular expression patterns from high-throughput data [1]. Over the last decade, biological pathways have provided natural sources of molecular mechanisms to develop diagnosis, treatment, and prevention strategies for complex diseases [2-4]. The various and massive functional genomics data are effectively analyzed by gene-set enrichment methods instead of individual gene analysis [5-8]. Pathway analysis and molecular signature discovery continue to reveal the association between genotypes and phenotypes, which are simply called molecular profiling or molecular phenotypes. At present, researchers intend to combine pathway and gene-set enrichment approaches and network module-based approaches to identify crucial relationships among different molecular mechanisms [1].

As sources of prior knowledge for molecular mechanisms, biological pathway databases are heterogeneous, cross multiple levels, and lack annotations [3]. Different pathway databases may yield divergent results from the same input data. When different databases yield similar results, applying multiple pathway data sources in a single analysis can generate a measure of validation. Unlike candidate pathway analysis, genome-wide pathway analysis does not require prior biological knowledge. In addition, genome-wide pathway analysis can reveal gene interactions across different diseases [3,9] and multiple pathways [3,10,11]. Other studies based on an online integrated human pathway database (HPD) also provided associations between different pathways with diverse types, sizes, and sources [12,13] on specific phenotypes. Although these efforts have greatly improved the efficiency of pathway analysis, our knowledge of biological pathways is still far from complete.

Gene signature data from the transcriptome level offers a complementary source of information to complete pathway knowledge. In a recent review, Khatri et al. [1] categorized pathway analysis into three generations of approaches: the first-generation "over-representation analysis" (ORA) approaches, the second-generation "functional class scoring" (FCS) approaches, and the third-generation "pathway topology" (PT) approaches. To overcome the limitations of ORA approaches (gene-level statistics), FCS approaches, such as gene-set enrichment analysis (GSEA) [6], were devised to include overall changes of gene expressions in each pathway/gene set (pathway-level statistics). Third generation approaches also include overall changes of gene expressions based on pathway topology--that is, their upstream/downstream positions within each pathway. Although these third generation approaches were meant to change our understanding of the underlying mechanisms of pathways, they lack information necessary to achieve this: the interdependence between pathways. Annotated knowledge from genome, transcriptome, post-transcriptome, and proteome levels can assist pathway and gene-set enrichment analysis.

Multi-level, multi-scale, knowledge-guided enrichment analysis can enable molecular phenotype discovery for specific human diseases. Currently, the acquisition of prior knowledge and systems modeling poses a challenge for developing tools that go beyond third-generation pathway analysis for disease-specific molecular profiling. Prior knowledge acquisition requires attention to updates and improves the available annotations with descriptive knowledge from multiple levels, especially for information on pathway microenvironment ("condition-, tissue-, and cell-specific functions of each gene") [1,3]. Systems biology modeling must incorporate data from the view of systems biology to build systems with multiple scales, which can be used to generate hypotheses that will give detailed and accurate predictions of changes in systems. Both aspects of this challenge will be addressed by building a database not only containing disease-associated genes, transcript factors, proteins, and microRNAs, but also by organizing their relationships within and between pathways, gene signatures, and any gene sets from existing experiments or papers.

To meet the new challenges of molecular phenotype discovery, we developed in this work an integrated online database, the Pathway And Gene Enrichment Database (PAGED), to enable comprehensive searches for disease-specific pathways, gene signatures, microRNA targets, and network modules, by integrating gene-set-based prior knowledge as molecular patterns from multiple levels--the genome, transcriptome, post-transcriptome, and proteome. The new database can provide the following benefits to biological researchers. First, the new database consists of disease-gene association data, curated and integrated from Online Mendelian Inheritance in Man (OMIM) [14] database and the Genetic Association Database (GAD) [15]; therefore, it has the potential to assist human disease studies. Second, as of March 2012 it also contains all current compiled gene signatures in Molecular Signatures Database (MSigDB) [8] and Gene Signatures Database (GeneSigDB) [7]. Third, it further integrates with microRNA-targets from miRecords [16] database, signaling pathways, protein interaction networks, and transcription factor/gene regulatory networks, partially based on data integrated from the Human Pathway Database (HPD) [12] and the Human Annotated and Predicted Protein Interaction (HAPPI) [17] database. All gene sets or pathways are annotated with molecular interaction details whenever available. We integrated the following version of the database OMIM [14] (Feb. 2012), GAD [15] (Aug. 2011), GeneSigDB [7] (v. 4.0, Sept. 2011), MSigDB [8] (v. 3.0. Sept. 2010), HPD [12] (2009), HAPPI [17](v. 1.4) and miRecords [16] (Nov. 2010), which are the latest versions available. An advantage of our work lies in its representation of relationships between pathways, gene signatures, microRNA targets, and/or network modules. These gene-set-based relationships can be visualized as a gene-set association network (GSAN), which provides a "roadmap" for molecular phenotype discovery for specific human diseases. Using colorectal cancer expression data analysis as a case study, we demonstrate how to query PAGED to discover crucial pathways, gene signatures, and gene network modules specific to colorectal cancer functional genomics.

## Methods

### Data sources

**Figure 1:**
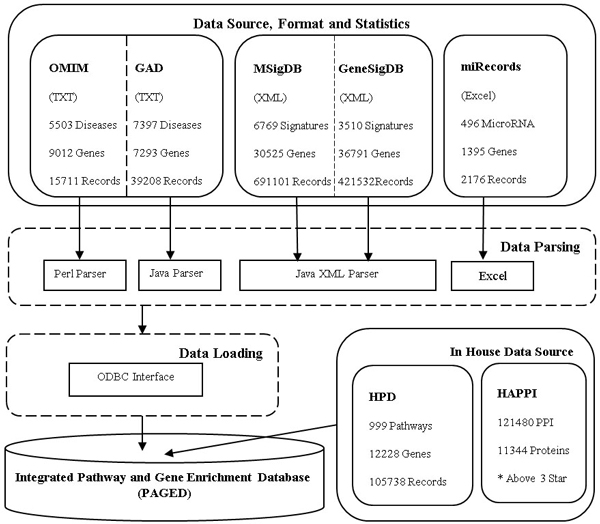
**An overview of the gene-set data integration process**. The figure shows the whole process of gene-set data integration and the basic statistics applied to gene-set data sources.

We show an overview of the data integration process in Figure 1. Gene-set data were collected, extracted, and integrated from five major categories. The pathway data sources were from HPD [12], which has integrated 999 human biological pathway data from five curated sources: KEGG, PID, BioCarta, Reactome, and Protein Lounge. The genome-level disease gene relationships were from OMIM [14] and GAD [15]; the transcriptome-level gene signatures were from MSigDB [8] and GeneSigDB [7]; the post-transcriptome-level microRNA data were from miRecords [16]; and the proteome level data was from an integrated protein interaction database HAPPI [17], which has integrated HPRD, BIND, MINT, STRING, and OPHID databases.

### Gene-set data integration

We treat as gene sets all groups of genes, including disease-associated genes, pathway genes, gene signatures, microRNA-targeted genes, and PPI sub-network modules. The raw files from those data sources have various formats including plaintext, XML, and table. We have written Perl/Java parsers to convert them into a common tab-delimited textual format to ensure syntactic-level data compatibility. To integrate across different databases, we mapped the gene/protein IDs in all databases to official gene symbols. The gene-set gene data is stored in our backend ORACLE11g relational database. As of the current release, PAGED contained a total of 25,242 gene sets, 61,413 genes, 20 organisms, and 1,275,560 records. All gene set members are represented by the official gene symbols. All PAGED gene sets were assigned unique PAGED-specific identifiers.

### Online software designing

The PAGED platform follows a multi-tiered design architecture. The backend was implemented as PL/SQL packages on an Oracle 11g database server and the PAGED application middleware was implemented on the Oracle Application Express (APEX) server, which bridged between the Apache webserver and the Oracle database server.

### Gene-set similarity measurement

Referring to the pathway similarity definition introduced in [12], the similarity score *S_i, j _*of two different gene sets is defined by the following formula:

(1) $$S_{i,j}=\alpha \times S_{L}+(1-\alpha )\times S_{R}=\alpha \times \frac{\left|P_{i}\cap^{}P_{j}\right|}{P_{i}\cup P_{j}}+(1-\alpha )\times \frac{\left|P_{i}\cap P_{j}\right|}{min\{\left|P_{i}\right|,\left|P_{j}\right|\}},(i\neq j)$$

Here, *P_i _*and *P_j _*denote two different gene sets, while |*P_i_*| and |*P_j_*| are the number of genes in each of these two gene sets. Their intersection *P_i_*∩*P_j _*denotes a common set of genes, while their union *P_i_*∪*P_j _*is calculated as |*P_i_*| + |*P_j_*| - |*P_i_*∩*P_j_*|. Here, *α *is a weight coefficient among [0, 1], which is used to count varying degree of contributions from calculations based both on the *overlap *(left item *S_L_*) and the *cover *(right item *S_R_*). *S_L _*is well-known as the Jaccard coefficient [18], which is often used to evaluate the similarity between two sets [19]. When a larger gene set covers a smaller one, we expect their similarity score to be high enough to identify them. In this situation, although the left item *S_L _*is a small number, the right item *S_R _*will be counted as 1.0 to make the final similarity score higher according to our definition in Equation (1), when taking an appropriate *α *value. Additional file 1 shows that how different *α *value could affect the distribution of the similarity scores of all cancer related gene sets. We found that when *α *fell in the interval of [0.7, 0.9], the score distribution would be close to a Poisson distribution. As we know, a Poisson distribution expresses the probability of a number of events occurring during a fixed period of time if these events occur with a known average rate and are time-independent since the last event. Therefore, we chose the middle value, *α *= 0.8, for the rest of the analysis. Our previous HPD paper [12] also validates the choice of 0.8 as the pathway similarity measurement.

### Microarray data

Here we use colorectal cancer (CRC) expression data analysis as a case study to show how to discover crucial pathways, gene signatures, and gene network modules specific to colorectal cancer functional genomics. We downloaded a colorectal cancer microarray dataset GSE8671 from Gene Expression Omnibus, GEO http://www.ncbi.nlm.nih.gov/geo/[20]. This microarray dataset compared the transcriptome data of 32 prospectively collected adenomas with those of the normal mucosa from the same individuals. Hence, we have 32 CRC samples and 32 normal samples. We used maximal expression values for the same proteins mapped from different Probe IDs, the Affy package in BioConductor for quantile normalization, the built-in MicroArray Suite (MAS5) for background correction, and Limma in BioConductor for differential analysis, the result of which is represented as fold changes (FC) of CRC samples vs. normal samples.

### Differential gene-set expressions

We use ABS_FC to denote the absolute value of fold change for each gene. We then define differential gene-set expressions here as

NORM_ABS_FC: The p*-norm of ABS_FC of all the available differential gene expressions in a gene set.

Usually, *p*-norm = ${\left(\sum_{\text{i}=1}^{\text{n}}{\left({\text{x}}_{\text{i}}\right)}^{\text{p}}\right)}^{\frac{1}{\text{p}}}$

For unification, we modify it as

(2) $$p^{*}\text{-norm}={((\frac{1}{\text{n}}\sum_{\text{i}=1}^{\text{n}}{\left({\text{x}}_{\text{i}}\right)}^{\text{p}}))}^{\frac{1}{\text{p}}}$$

In the implementation, *p *= 6 performs the best at accentuating highly differential expressions in a gene set.

### Gene-set association network (GSAN) construction

To visualize the relationships between gene sets, we define a gene-set association network (GSAN) as a network of associations between different gene sets, in which the network element representation is as follows:

• Node: Gene set

• Edge: Association between two gene sets

• Node size: Gene-set scale (Counting genes in each gene set)

• Node color: Differential gene-set expression (NORM_ABS_FC)

• Node line color: Gene-set data source

• Edge width: Similarity score

## Results

### Database content statistics

Table 1 lists the detailed statistics for each data source and the overlap between each pair. For example, MSigDB contains 30,525 genes and GeneSigDB contains 36,791 genes. The number of overlapping genes between these two databases is 17,209. We found a synergistic effect from integrating these two signature databases, resulting in greatly increased gene-set coverage. The same effect was observed for all the remaining pair comparisons. These data sources proved to be complementary.

**Table 1:** Number of overlapping genes between different data sources

	OMIM	GAD	MSigDB	GeneSigDB	miRecords	HPD	HAPPI*
OMIM	9012	1862	3489	2792	231	2559	3849
GAD		7293	6821	6450	432	3202	4922
MSigDB			30525	17209	759	6229	10677
GeneSigDB				36791	900	5904	10395
miRecords					1395	443	725
HPD						12228	10512
HAPPI							21955

* Only PPIs of over 3-star quality are considered here; to calculate the overlap, protein IDs from HAPPI have been first converted to gene symbols.

### Gene-set scale distributions

**Figure 2:**
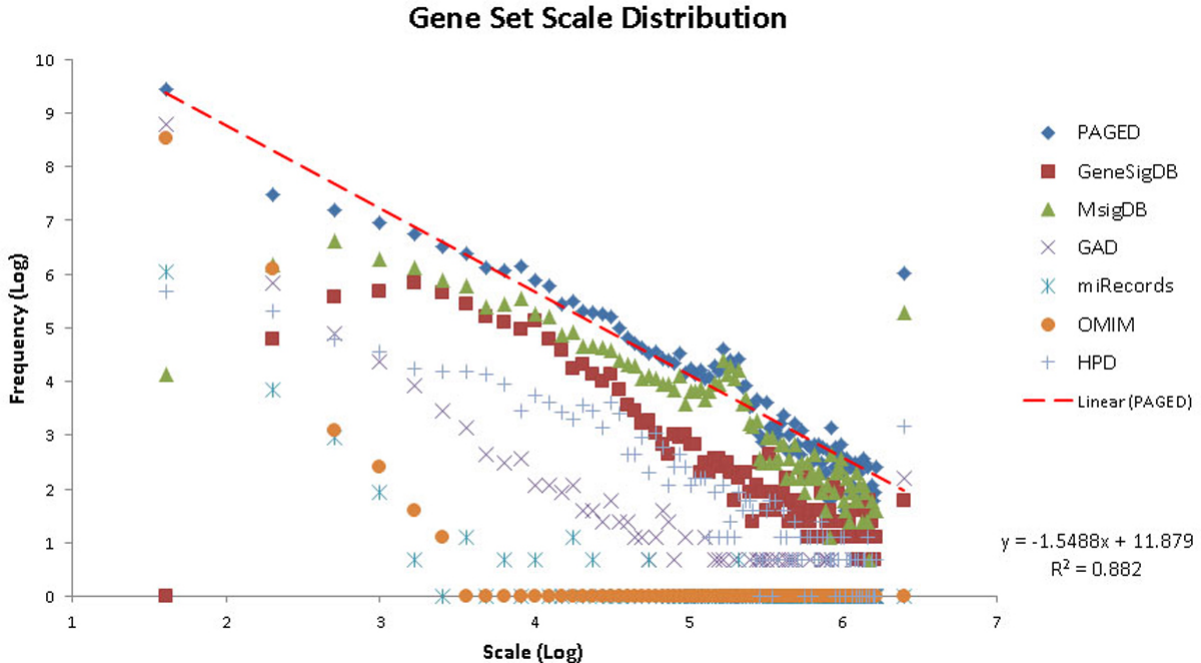
**Gene-set scale distributions for PAGED molecule data**. A gene-set scale refers to the number of molecules (i.e., genes) involved in a given gene set. The frequency on the y-axis refers to the count of all gene sets falling into the category of a particular gene-set scale size on the x-axis. The distributions are plotted under log scale for both the x-axis and y-axis. The linear trend line in red represents linear regression of PAGED distribution and the linear equation and its R-Square are listed.

The gene-set scale can reflect the integrality of information content of a biological topic. In this study, we define gene-set scale as the number of molecules (i.e., gene symbols) in a gene set. We performed a statistical analysis of the gene-set scale distributions of both PAGED and of its individual data sources. Figure 2 shows that each data source taken by itself is not very scale-free, especially for OMIM, GAD, and miRecords for higher scales and HPD, GeneSigDB, and MSigDB for lower scales. The scale distribution of PAGED is relatively scale-free on both the low end and the high end with a linear regression *R*-squared of 0.88. Additionally, the distribution of PAGED always lies above those of its data sources, indicating that the integration has enriched the number of gene sets in all scales instead of exhibiting a bias towards one particular scale. These observations indicate that the integration process of PAGED has resulted in a database that can take account of different gene set scales.

### Online functionalities

**Figure 3:**
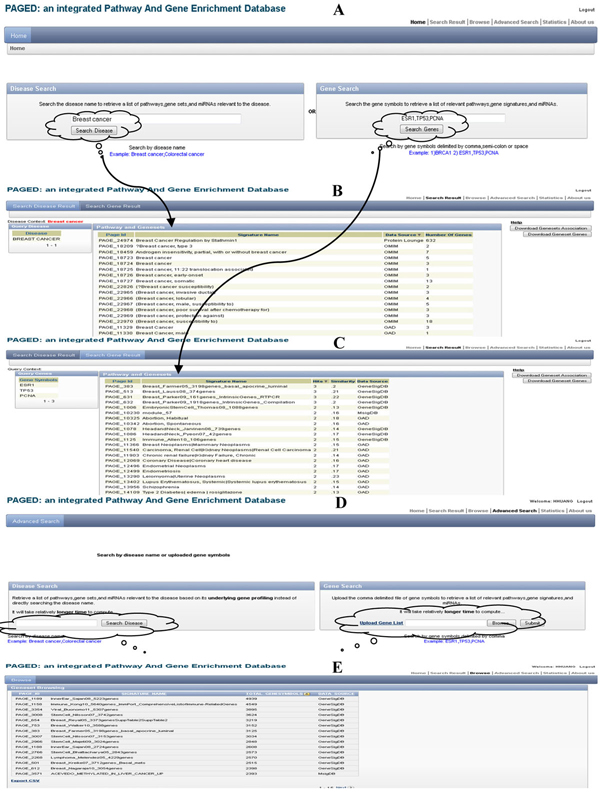
**An overview for the core functionality of the online PAGED website**. (A) The PAGED home page providing search by either disease name or gene list; (B) a webpage containing the list of gene sets retrieved as a result of a disease query; (C) a webpage containing the list of gene sets retrieved as a result of a gene list query; (D) an advanced search page in which the user can either search disease name or upload a gene-list to search; (E) a browse page listing the gene sets, their data source and number of genes.

In Figure 3, we show the user interfaces of the PAGED website. It supports both disease-based search and user-defined gene-list search. If users search the disease term in the home page (Figure 3A), PAGED will retrieve a list of related gene sets by directly matching the disease term with all the gene-set names; if users instead search a disease term in the advanced search page (Figure 3D), PAGED will first retrieve disease-relevant genes from OMIM and GAD and then use those genes to query the whole database, which will retrieve a gene-set list based on disease gene profiles that is more comprehensive than that of either OMIM or GAD individually. Users can also search PAGED using multiple genes in the home page (by delimiting them with a comma) to retrieve a list of related gene sets with the hits number and similarity scores (Figure 3A). In addition, users can upload a file of their genes with one gene per line on the advanced search page (Figure 3D) to perform the gene-based search. In the advanced gene-based search (Figure 3D), user can also perform an organism specific search though the majority of the gene-sets are human related. All the gene sets are hyperlinked to the original database, where user can further examine the detailed annotations of that specific gene set.

Upon executing the queries, PAGED can retrieve a list of related gene sets in an HTML table (Figure 3B, C) with their specific organism information included, which are downloadable as a comma-separated value (CSV) file. On the same page, there are links for downloading all the genes in those gene sets and the association between each gene set. In the gene set association downloading page, a simple heat map is provided for the visualization of gene set similarities. More sophisticated visualization will be provided in the near future.

### Case studies

The following case studies use colorectal cancer expression data analysis as a case study to demonstrate how to discover crucial pathways, gene signatures, and gene network modules specific to colorectal cancer functional genomics.

#### Case study I: Searching disease-associated gene sets based on gene-set names

Using the standard query box provided at the PAGED home page, one can search for *colorectal cancer *in all biological gene sets. PAGED returns a list of gene sets, which can be ordered by decreasing number of genes contained by each gene set. In total, 45 gene sets from three data sources (i.e., OMIM, GAD and KEGG) have been retrieved. Not surprisingly, most of them are disease-related gene sets from either OMIM or GAD. Only 1 (i.e., "Colorectal cancer pathway") out 45 is from KEGG. The top 10 search results are listed in Table 2.

**Table 2:** Top 10 search results by querying colorectal cancer at the home page

Gene-set Name	# of Genes	Data Source
colorectal cancer	433	GAD
Colorectal cancer	134	KEGG
Colorectal cancer	14	OMIM
Colorectal cancer, somatic	12	OMIM
Colorectal cancer, hereditary non-polyposis, type 8	7	OMIM
Colorectal cancer, susceptibility to	7	OMIM
Colorectal cancer, hereditary non-polyposis, type 6	6	OMIM
Breast and colorectal cancer, susceptibility to	5	OMIM
Colorectal Cancer	5	GAD

#### Case study II: Searching disease-associated gene sets based on gene-set components

Next, a user can search with the same term *colorectal cancer *on the advanced search page, which uses the disease's gene profile to search for gene sets. PAGED first obtained 203 colorectal cancer related genes from OMIM and GAD. Then, it used those genes to retrieve a total of 4,932 gene sets with at least 2 hits. Since we are more interested in gene sets other than disease terms, we excluded those gene sets from OMIM and GAD for further analysis. To rule out the possibility that those gene sets were hit randomly, we did a Fisher's exact test to calculate the *p*-value between those 203 genes and every retrieved gene set. Finally, we obtained 3,879 gene sets with a *p*-value < 0.05 and hits ≥2. These gene sets are from all data sources, including MSigDB, GeneSigDB, miRecords, and all pathway data sources from HPD. Both the number of gene sets and their variety support the conclusion that advanced disease search based on gene profiles are more comprehensive than a simple disease search.

Table 3 shows the top results ranked by decreasing number of hits from each data source. Protein Lounge suggests "Molecular Mechanisms of Cancer," "Akt Signaling," and other important pathways in colorectal cancer; BioCarta suggests "wnt signaling pathway"; and NCI Nature curated suggests "Canonical Wnt signaling pathway." These are all very important pathways in colorectal cancer development [21]. Similarly, "Colorectal cancer" and "p53 signaling pathway" from KEGG, "SIGNAL_TRANSDUCTION" and "KEGG_PATHWAYS_IN_CANCER" from MSigDB, and cancer-related signatures/microRNA from GeneSigDB/miRecords from Table 3 reveal a comprehensive picture of the important gene sets involved in colorectal cancer. Thus, the results of the advanced search yield more insights about colorectal cancer mechanisms than those of the simple search.

**Table 3:** Top search results of *colorectal cancer *advanced search

Gene-set Name	Hits	P value	FDR	Data Source
Molecular Mechanisms of Cancer	38	2.48E-17	7.04E-10	Protein Lounge
PI3K Signaling	33	2.01E-13	7.04E-10	Protein Lounge
Akt Signaling	27	9.6E-13	7.04E-10	Protein Lounge
ERK Signaling	24	1.53E-10	7.04E-10	Protein Lounge
GSK3 Signaling	23	1.32E-13	7.04E-10	Protein Lounge
inactivation of gsk3 by akt causes accumulation of b-catenin in alveolar macrophages	9	3.7E-11	7.04E-10	BioCarta
atm signaling pathway	8	6.28E-11	7.04E-10	BioCarta
wnt signaling pathway	7	7.7E-09	7.04E-10	BioCarta
cell cycle: g2/m checkpoint	7	2.14E-08	7.04E-10	BioCarta
cell cycle: g1/s check point	7	2.14E-08	7.04E-10	BioCarta
Canonical Wnt signaling pathway	8	9.24E-10	7.04E-10	NCI-Nature
Presenilin action in Notch and Wnt signaling	8	3.16E-08	7.04E-10	NCI-Nature
Plasma membrane estrogen receptor signaling	7	1.41E-08	7.04E-10	NCI-Nature
FOXM1 transcription factor network	7	2.48E-07	7.04E-10	NCI-Nature
LPA receptor mediated events	7	1.45E-06	7.04E-10	NCI-Nature
Metabolism of xenobiotics by cytochrome P450	20	3.3E-25	7.04E-10	KEGG
Drug metabolism - cytochrome P450	17	4.96E-21	7.04E-10	KEGG
Bladder cancer	15	3.29E-18	7.04E-10	KEGG
Cytokine-cytokine receptor interaction	15	1.39E-06	7.04E-10	KEGG
Colorectal cancer	14	4.43E-14	7.04E-10	KEGG
p53 signaling pathway	14	4.92E-14	7.04E-10	KEGG
Prostate cancer	14	1.66E-12	7.04E-10	KEGG
Xenobiotics	5	3.32E-08	7.04E-10	Reactome
Formation of incision complex in GG-NER	5	5.75E-06	7.04E-10	Reactome
Global Genomic NER (GG-NER)	5	5.75E-06	7.04E-10	Reactome
Dual incision reaction in GG-NER	5	5.75E-06	7.04E-10	Reactome
Exocytosis of Alpha granule	5	0.000217	1.95E-08	Reactome
SIGNAL_TRANSDUCTION	55	8.36E-28	7.04E-10	MsigDB
BIOPOLYMER_METABOLIC_PROCESS	49	4.16E-22	7.04E-10	MsigDB
KEGG_PATHWAYS_IN_CANCER	43	9.9E-46	7.04E-10	MsigDB
NUCLEOBASENUCLEOSIDENUCLEOTIDE_AND_NUCLEIC_ACID_METABOLIC_PROCESS	41	2.16E-20	7.04E-10	MsigDB
NUCLEUS	41	1.8E-18	7.04E-10	MsigDB
Immune_Kong10_5640genes_ImmPort_ComprehensiveListofImmune-RelatedGenes	114	3.61E-49	7.04E-10	GeneSigDB
Lymphoma_Melendez05_4229genes	81	1.57E-39	7.04E-10	GeneSigDB
Breast_Farmer05_3198genes_basal_apocrine_luminal	66	1.08E-21	7.04E-10	GeneSigDB
Ovarian_Crijns09_2394Genes_17PathwayPredictor	57	7.94E-30	7.04E-10	GeneSigDB
StemCell_Nilsson07_3742genes	45	4.86E-07	7.04E-10	GeneSigDB
hsa-miR-19a	3	1.49E-05	8.43E-09	miRecords
[hsa-miR-21]	3	0.000116	8.43E-09	miRecords
hsa-miR-204	3	0.000164	1.95E-08	miRecords
hsa-miR-21	3	0.000953	2.72E-07	miRecords
hsa-miR-125b	3	0.003089	2.72E-07	miRecords

#### Case study III: Searching gene sets similar to user-defined query gene sets

To use the gene-based search from PAGED, we first analyzed a colorectal cancer microarray dataset GSE8671 with BioConductor to identify the differential genes. We selected the top 100 genes ranked by the absolute fold change with *p*-values less than 0.05. After querying PAGED with those 100 genes, we obtained 1,707 gene sets, out of which 1,152 also satisfied Fisher's exact test of a *p*-value less than 0.05. Those gene sets span from all the data sources except BioCarta and miRecords. Table 4 lists the top results ranked by the number of hits. Most of them are cancer-related gene sets. Specifically, "SABATES_COLORECTAL_ADENOMA_DN" and "SABATES_COLORECTAL_ADENOMA_UP" from MSigDB and "Intestine_Vecchi07_1024genes" and "Colon_Kim04_235genes" from GeneSigDB supports the importance of those 100 query genes to colorectal cancer. This case study also shows the complementary nature of MSigDB and GeneSigDB and thus the benefit of integrating them, which has also been proved by [22]

**Table 4:** Top search results of gene-based search from microarray datasets

Gene-set Name	Hits	P value	FDR	Data Source
SABATES_COLORECTAL_ADENOMA_DN	58	4.57E-96	2.76E-10	MsigDB
Breast_Farmer05_3198genes_basal_apocrine_luminal	35	2.91E-13	2.76E-10	GeneSigDB
SABATES_COLORECTAL_ADENOMA_UP	34	1.62E-57	2.76E-10	MsigDB
Immune_Kong10_5640genes_ImmPort_ComprehensiveListofImmune-RelatedGenes	34	3.56E-08	2.76E-10	GeneSigDB
Leukemia_Pellegrini08_2692genes	32	1.28E-15	2.76E-10	GeneSigDB
Intestine_Vecchi07_1024genes	28	3.91E-23	2.76E-10	GeneSigDB
Viral_Buonomo11_5307genes	25	6.45E-05	0.000109	GeneSigDB
SMID_BREAST_CANCER_LUMINAL_B_DN	23	4.16E-19	2.76E-10	MsigDB
Lymphoma_Melendez05_4229genes	22	2.03E-06	2.76E-10	GeneSigDB
Colon_Kim04_235genes	21	5.18E-30	2.76E-10	GeneSigDB
Breast_Parker09_1918genes_IntrinsicGenes_Compilation	21	1.18E-08	2.76E-10	GeneSigDB

#### Case study IV: Building disease-specific gene-set association networks (GSANs) based on gene-set similarities

**Figure 4:**
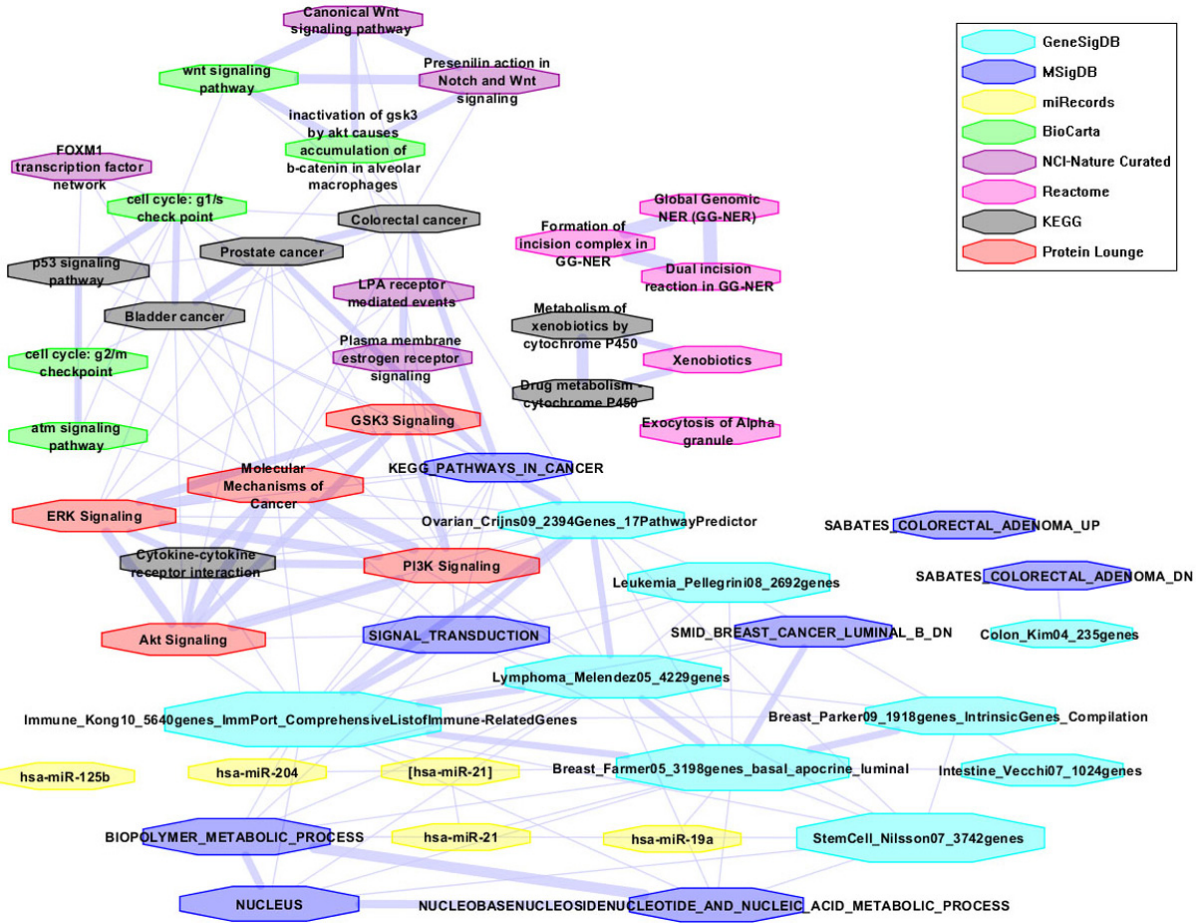
**CRC-specific gene-set association network (GSAN) on the top gene sets from colorectal cancer study**. Node size: Gene-set scale (Counting genes in each gene set); Node color: Gene-set data source; Edge width: Similarity score (≥ 0.1). All gene sets are highly connected to each other, suggesting their collaborative functions in colorectal cancer.

With the unique top 50 gene sets related to colorectal cancer from disease search and gene search (Table 3 and Table 4), we next investigated the gene-set associations between them; 863 associations were found by overlapping the gene symbols between each pair of gene sets, out of which 642 also satisfied Fisher's exact test of a *p*-value and FDR less than 0.05. A network visualization using Cytoscape [23] is shown in Figure 4. Most of those gene sets are connected to one another, and a few share a large number of genes, suggesting that they form a collaborative unit in colorectal cancer.

#### Case study V: Prioritizing disease-associated gene sets by using differential gene-set expressions

**Figure 5:**
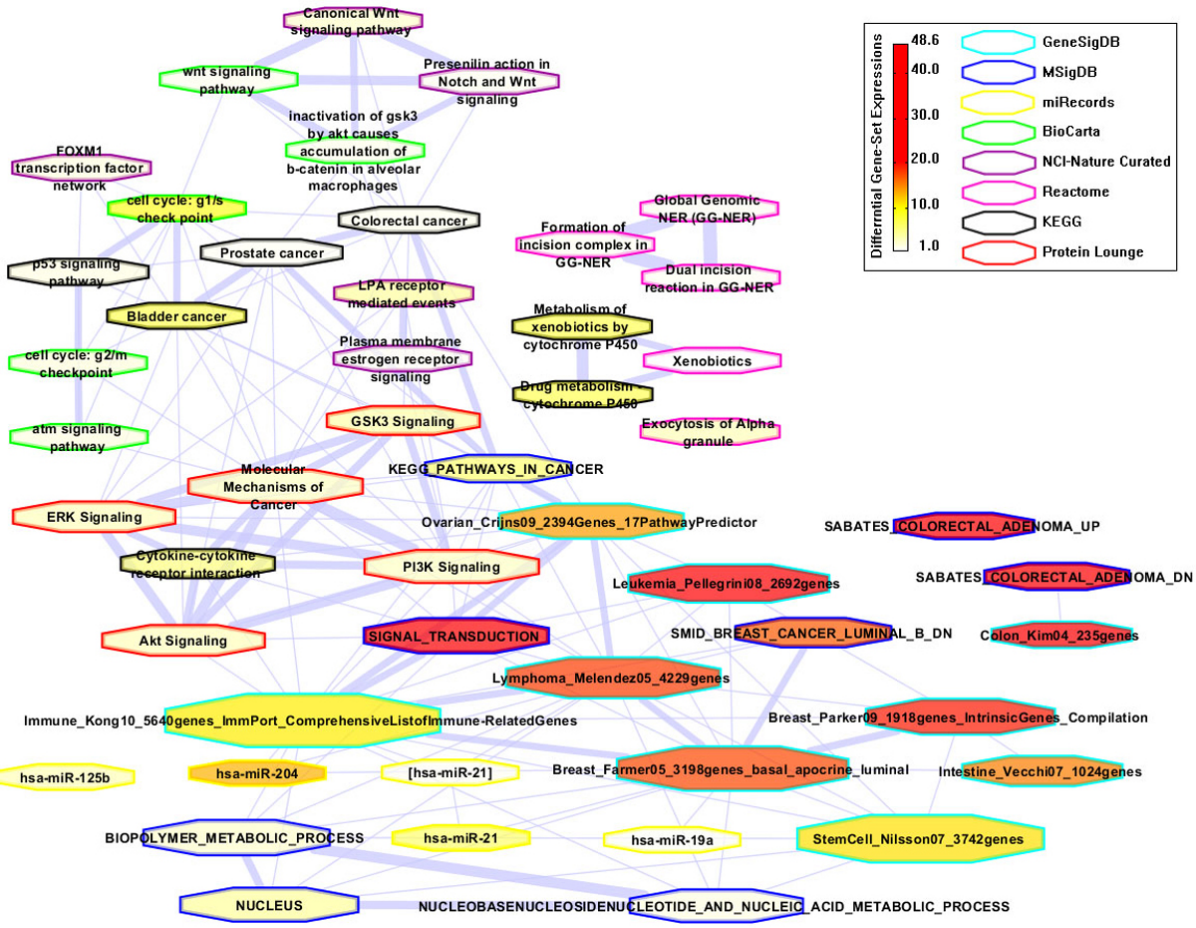
**CRC-specific gene-set association network (GSAN) with differential gene-set expressions**. The differential gene expressions are from the differential analysis based on the microarray data, GSE8671. Node size: Gene-set scale (Counting genes in each gene set); Node color: Differential gene-set expression (NORM_ABS_FC); Node line color: Gene-set data source; and Edge width: Similarity score (≥ 0.1).

First, the differential gene expression value (ABS_FC) for each gene in a gene set is calculated from the differential analysis based on the microarray data GSE8671. Second, the differential gene-set expression value (NORM_ABS_FC) for each gene set in the CRC-specific GSAN is calculated by using Equation (2). Third, a CRC-specific GSAN with differential gene-set expressions is shown in Figure 5, in which node size represents gene-set scale (Counting genes in each gene set); node color represents differential gene-set expression (NORM_ABS_FC); node line color represents the gene-set data source; and edge width represents the similarity score. By considering differential gene-set expressions for each gene set, we prioritize top-selected gene sets as shown in Table 5. Most of top-ranked gene sets are closely related to colon tissue, colorectal cancer, or other cancers, which implies that our database can not only support comprehensive disease-associated gene-set searching and browsing, but also accurate, disease-specific gene-set prioritizing by using the concept of differential expressions at the gene-set level.

**Table 5:** Top 20 gene sets ranked by differential gene-set expressions in the CRC-specific gene-set association network (GSAN)

Gene-set name	Scale	Data Source	NORM_ABS_FC
Colon_Kim04_235genes	151	GeneSigDB	48.58225017
SABATES_COLORECTAL_ADENOMA_DN	292	MsigDB	43.9233159
SIGNAL_TRANSDUCTION	1598	MsigDB	32.5957784
Leukemia_Pellegrini08_2692genes	2122	GeneSigDB	31.65148925
SABATES_COLORECTAL_ADENOMA_UP	142	MsigDB	31.65000681
Breast_Parker09_1918genes_IntrinsicGenes _Compilation	1734	GeneSigDB	20.85621131
Lymphoma_Melendez05_4229genes	2570	GeneSigDB	19.38449282
Breast_Farmer05_3198genes_basal _apocrine _luminal	3125	GeneSigDB	18.93820407
SMID_BREAST_CANCER_LUMINAL_B_DN	648	MsigDB	18.13762096
Intestine_Vecchi07_1024genes	796	GeneSigDB	16.68882931
Ovarian_Crijns09_2394Genes _17PathwayPredictor	1586	GeneSigDB	15.29529767
hsa-miR-204	19	miRecords	14.37015815
StemCell_Nilsson07_3742genes	3624	GeneSigDB	12.47045771
Immune_Kong10_5640genes_ImmPort _ComprehensiveListofImmune-RelatedGenes	4549	GeneSigDB	11.91186233
cell cycle: g1/s check point	53	BioCarta	9.84279867
Bladder cancer	89	KEGG	7.885181064
Drug metabolism - cytochrome P450	94	KEGG	7.837851592
Metabolism of xenobiotics by cytochrome P450	103	KEGG	7.837805455
hsa-miR-21	34	miRecords	7.001844224
KEGG_PATHWAYS_IN_CANCER	328	MsigDB	6.792625895

## Discussion

In the near future, we will improve gene-set similarity algorithms by using a global PPI network to calculate their distance. This would provide a more robust measurement for web interface development, and we plan to add a disease browsing function based on disease ontology and a network visualization function to show the gene-set association dynamically. Our final goal is to perform multi-scale network modeling for molecular phenotype discoveries by integrating differential expressions with pathway and network topologies. The current release of PAGED provides a solid foundation for us to develop third-generation pathway analysis tools [1].

## Conclusions

We developed PAGED, an online database that provides the most comprehensive public compilation of gene sets. In the current release, PAGED contains a total of 25,242 gene sets, 61,413 genes, 20 organisms, and 1,275,560 records from five major categories: the pathway data from HPD, genome-level disease data from OMIM and GAD, transcriptome-level gene signatures from MSigDB and GeneSigDB, the post-transcriptome microRNA data from miRecords, and proteome-level data from HAPPI. The number of overlapping genes between each data source, gene-set scale distribution, and case study in colorectal cancer shows the synergistic effect of integrating data sources, which greatly facilitate access to gene-set-based prior knowledge. The current PAGED software can help users address a wide range of gene-set-related questions in human disease biology studies.

## Competing interests

The authors declare that they have no competing interests.

## Authors' contributions

JYC conceived of this work, guided the research team by providing ideas and feedback along the way, and revised the manuscript. HH integrated disease-gene association data, developed the website, designed the case studies and wrote the manuscript. XW participated in the idea initiation, framework development, data quality control, case studies, and manuscript writing. MS integrated various pathways, microRNA, and gene signature data. SNM reviewed the evolvement on pathway analysis and gene-set enrichment analysis. RP helped with the database management and maintenance. KFM tested the website, provided valuable suggestions for substantial improvements, and revised the manuscript. PW assisted with website maintenance. All authors read and approved the final manuscript.

## Supplementary Material

Additional file 1 **Change of similarity score with different *α *in Equation I**. The frequency on the y-axis refers to the count of all gene set pairs falling into the category of a particular similarity range on the x-axis. Different *α *in Equation I have been used to calculate the similarity score. When *α *approaches 0, the distribution skewed to right with many false positive high similarity scores; when *α *approach 1, the distribution is too left-skewed failing to differentiate those low similarity scores apart. Click here for file
