# Supplementary figures and images for: PAGED: a pathway and gene-set enrichment database to enable molecular phenotype discoveries
Source: BMC Bioinformatics. 2012 Sep 11;13(Suppl 15):S2. doi: 10.1186/1471-2105-13-S15-S2 (PMC3439733; doi:10.1186/1471-2105-13-S15-S2)

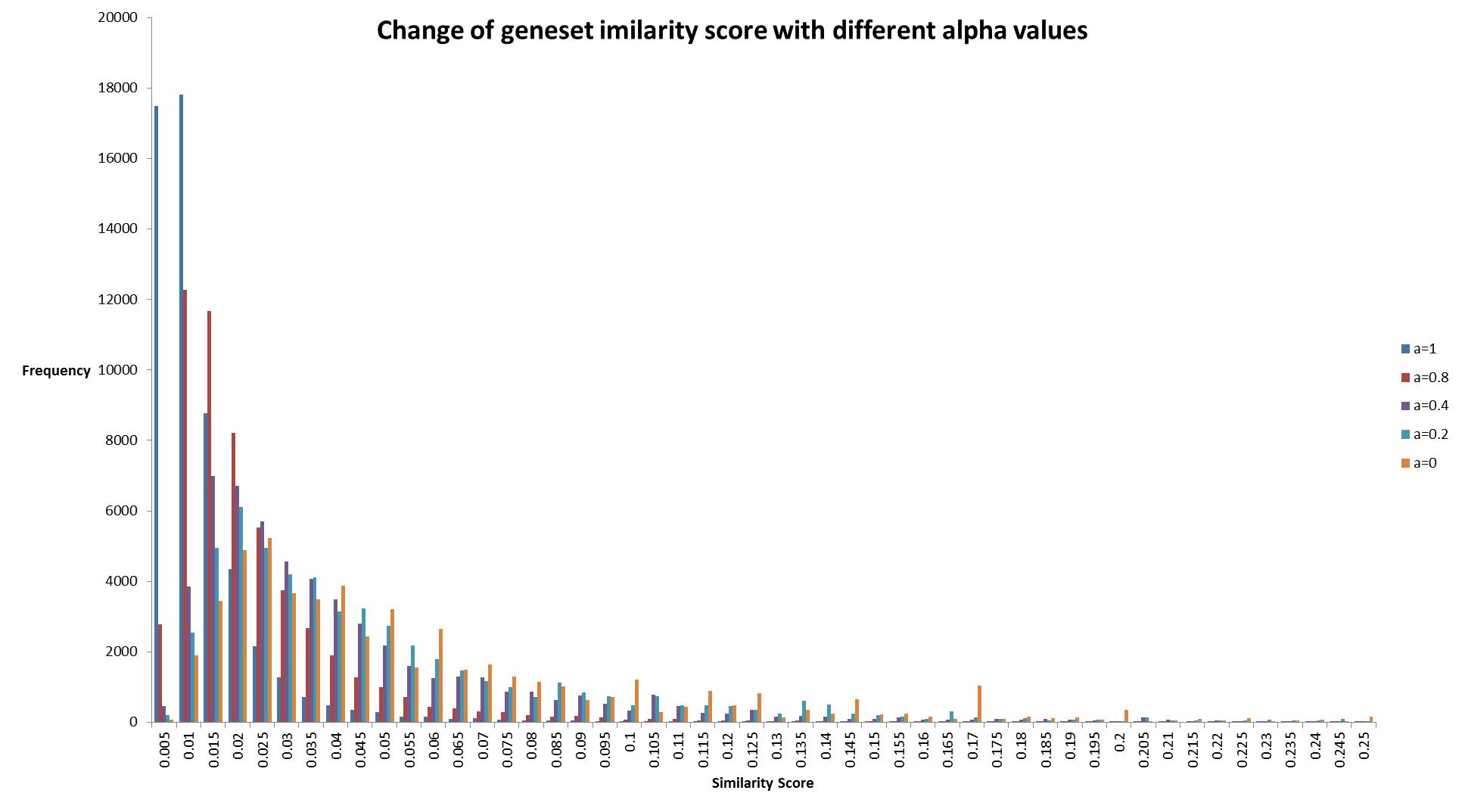

Supplement: Additional file 1 — Change of similarity score with different α in Equation I. The frequency on the y-axis refers to the count of all gene set pairs falling into the category of a particular similarity range on the x-axis. Different α in Equation I have been used to calculate the similarity score. When α approaches 0, the distribution skewed to right with many false positive high similarity scores; when α approach 1, the distribution is too left-skewed failing to differentiate those low similarity scores apart. [file 1471-2105-13-S15-S2-S1.jpg]
